# A follow-up on patients with severe mental disorders in Sardinia after two changes in regional policies: poor resources still correlate with poor outcomes

**DOI:** 10.1186/1471-244X-13-333

**Published:** 2013-12-06

**Authors:** Mauro Giovanni Carta, Matthias C Angermeyer, Federica Sancassiani, Francesco Tuligi, Roberto Pirastu, Anna Pisano, Elisa Pintus, Gisa Mellino, Mirra Pintus, Emanuele Pisanu, Maria Francesca Moro, Davide Massidda, Giuseppina Trincas, Dinesh Bhugra

**Affiliations:** 1 Department of Public Health, Clinical and Molecular Medicine, University of Cagliari, via Ospedale, 117 Cagliari, Italy; 2 Department of Mental Health (DSM)- of Lanusei, Lanusei, Italy; 3 Department of Mental Health (DSM)- of Carbonia-Iglesias, Carbonia, Italy; 4 Department of Mental Health (DSM)- of Cagliari, Cagliari, Italy; 5 HSPRD Institute of Psychiatry (KCL) London, London, UK; 6 Centre for Public Mental Health, Gösing am Wagram, Austria

**Keywords:** Quality of care, Mental health, Psychosis, Follow-up, Social functioning, Resources

## Abstract

**Background:**

This survey followed a cohort of patients with chronic psychosis recruited from five catchment areas (DSMs) of the Sardinian community mental health services. The objective was to examine whether the amount of resources in the different sites may be a determinant of the outcomes.

**Methods:**

Naturalistic follow-up study on 309 consecutive users with diagnosis of schizophrenic disorder, schizoaffective disorder, bipolar affective disorder with psychotic symptoms (DSM-IV TR) of five Sardinian community mental health services. Mental state and clinical symptoms along with functioning were assessed using semi-structured clinical interviews (ANTAS), Clinical Global Impression Severity Scale (CGI-S), Global Assessment of Functioning Scale (GAF) and Health of the Nation Outcome Scales (HONOS). Assessments were conducted at the beginning of the study and after one year.

**Results:**

The proportion of professionals working in all DSMs participating in the study was found lower than the national Italian standard (0.7 vs 1.0 per 1,500 inhabitants). Follow-up revealed significant differences between DSMs in the improvement of the Honos scores (F = 5.932, p = 0.000). These differences correlate with the improvement of resources in terms of number of professionals during, and one year prior, to the trial.

**Conclusions:**

The study shows that mental health services provided in the public sector in Sardinia are still very resource-poor, at least in terms of human resources. Our findings suggest that mental health service resources influence outcomes as regards the social functioning of users. We urge policy makers to take these observations into account when planning future services.

## Background

The reform of mental health care in Italy, triggered off by the so-called “Basaglia Law”, attracted much attention worldwide [1]. The closure of psychiatric hospitals signified a major shift from the traditional asylum-based models of care, but community mental health services did not develop at a similar level across the country. Not surprisingly, the greatest difficulties occurred in the initial phase especially in the southern regions. Only subsequently did some southern regions of Italy implement complex networks of care [2]. Thus implementation of the psychiatric reform was accomplished at the end of the 1990s, which marked the end of the state mental hospital system in nationwide Italy [3].

However, innovative forms of mental health care have not always been adequately investigated or evaluated using standards of epidemiological research and quality assessment. Thus, the debate about the “right” model of psychiatric care has often been ideological rather than based on sound health service research and data [4].

Sardinia was one of those Italian regions in which the reform was implemented late [5]. Ten years following the entry into force of the famous “Law 180” it was clear that the delay in building a community care system had led to severe negative consequences for patients with severe psychiatric disorders, such as an increase in the "revolving door" phenomenon in psychiatric units in general hospitals (which should focus on the management of emergencies only), a very high average length of hospitalization and an excess of suicides in patients discharged from psychiatric hospitals, who showed a higher risk for suicide than the general population living in the same region as well as a higher standardized mortality ratio for suicide in comparison to patients discharged from hospitals in other areas of Italy where a comprehensive community mental health care system had been implemented. Finally, a very high rate of compulsory admissions to psychiatric units [5,6] was found. The year 2005 was a noteworthy turning point in Sardinian regional policies. At that time a model inspired by ideas promulgated by psychiatrists from Trieste was introduced and adapted. Hence, the network of care was restructured stressing assertive treatment of the severely ill by keeping community care facilities open 24 hours a day, providing beds for patients within the community care structure and emphasizing a social/work inclusion model. This was in marked contrast to a more medically oriented model.

Inevitably this change of pace in policy sparked a fierce debate among those who were opposed to the regional reform, including the Italian Society of Psychiatry through the voice of its president and its regional coordinator, as expressed in press interviews during a national congress held in Sardinia at the time [7] and those who were in favor of the reform such as associations of families with mentally ill people [8]. However, neither side in this debate was able to provide empirical evidence supporting their respective arguments. In 2008, there was a political change in the government of the region: the new administration, led by the previous opposition political party, decided to radically change the guidelines of policy in health management and that the reform of the mental health care should cease. In opposition it introduced a more traditional system of mental health care in 2009.

Prior to these heated regional debates, which lasted from 2007 to 2010, a few studies have compared the well-being, health and social needs of mental health patients in the care of Sardinian Public Health Facilities with those of other Italian and European regions. However a European study conducted prior to this debate showed that patients with chronic schizophrenia treated at two centers of public mental health in Sardinia, followed over a period of one year, were those with the largest number of un-met social needs among all collaborating centers in Europe [9].

The main objective of this study was to carry out a follow-up study on patients with psychosis who were being cared for by the public community mental health system in Sardinia, using measures of outcome allowing the comparison with other similar international and national studies. The study was carried out during 2010–2011, just at the end of the reform period. A secondary objective was to investigate whether the amount of resources available, expected to be sufficient given the extent of the political debate on mental health care, was a determinant of the social and clinical outcomes in this patient cohort.

## Methods

### The regional public mental health care system in Sardinia and the collaborating centers

The Regional Public Mental Health Care System is organized in Sardinia on the basis of eight similar sector areas (*Dipartimenti di Salute Mentale* - DSM) corresponding to the eight administrative areas (“*Province*”). Each administrative area is divided into community catchment areas (*Centri di Salute Mentale* – CSM) serving a target adult population between 50.000 and 150.000 inhabitants. The amount of staff resources available for the various catchment areas were assessed at the time of the study and findings are presented elsewhere [10].

Five out of eight Administrative Mental Health Catchment Areas (DSMs) took part in the study. From DSM 1, one out of three CSMs were included in the study, from DSM 4 the only existing CSM, from DSM 6 and 7 one out of two CSMs, and from DSM 8 three out of eight CSMs.

### Design and sample

A naturalistic follow-up study was conducted with a follow-up period of one year. Assessments were made at the start (T0) and at the end of the study (T1).

In each participating CSM all patients with the diagnosis of schizophrenic disorder, schizoaffective disorder or bipolar affective disorder with psychotic symptoms (DSM-IV TR) who had been treated by the CSM at least during the previous two years (with at least three contacts per year in the last two years) were recruited.

Those patients fulfilling the inclusion criteria who had contacted the centers during the same index week were recruited in the cohort. In total, 309 patients were enrolled in the study.

### Instruments

The psychiatric diagnosis was made according to DSM-IV TR [11]. by clinicians using ANTAS, which is a semi-structured interview [12,13] previously successfully used and validated in this population.

The level of functioning was measured using the well-known and well-validated Global Assessment of Functioning (GAF) scale [11]. The scale scores range from 0 to 100, with higher scores indicating higher levels of functioning.

Illness severity was assessed using the Clinical Global Impression – Severity scale (CGI-S) [14]. The CGI-S is a 7-point Likert scale that requires the clinician to rate the severity of the patient's illness at the time of assessment. Based on his/her global clinical impression, the clinician assesses the patient as: 1 = “normal, not at all ill”; 2 = “borderline mentally ill”; 3 = “mildly ill”; 4 = “moderately ill”; 5 = “markedly ill”; 6 = “severely ill”; 7 = “extremely ill”.

Health and social functioning were assessed with the help of the Health of the Nation Outcome Scale (HoNOS) [15]. The HoNOS measures patient outcomes in four main domains: behavior, cognitive and physical impairment, symptoms and social functioning. Each item is scored from 0 to 4, ranging from 0 = “no problem” to 4 = “severe/very severe problem”.

Moreover, demographic and clinical variables (age, sex, marital status, parenthood status, housing, employment status, education, time in treatment by the mental health service, current and previous treatments), as well as other process and structure indicators were assessed by means of an ad hoc developed instrument.

Data on human resources were collected from administrative records of the centers and compared with the standards recommended by the Italian Ministry of Health, according to which staff should include at least 1 professional (including doctors, psychologists, social workers, nurses and administrative professionals) for a target population of 1,500 inhabitants [16].

### Assessment

Data were assessed at the start of the study (T0) and after one year (T1). The assessment was made by independent raters who did not belong to the staff of the centers. They used all the sources needed for completing the different instruments (patients, professionals and clinical records). All raters underwent one week of intensive training in the use of the instruments, with crossed assessments to improve the reliability of their assessments.

### Statistical analysis

To describe the main socio-demographic and clinical characteristics of the sample, the kind of mental health services used and the resources involved, we conducted a series of descriptive analyses (frequency and percentage for categorical variables; mean and standard deviation for continuous variables).

To test the correlation between outcome indicators we performed Pearson correlations at T0 and T1.

To test the differences between DSMs on the CGI-S, GAF and HoNOS total scores at T0, we conducted a multivariate analysis of variance (MANOVA), with DSMs as the between-subjects factor.

To test the amount of improvement in outcome measures, we computed the difference between scores obtained at T0 and T1 (ΔT0-T1): a positive ΔT0-T1 indicates an improvement, a negative ΔT0-T1 a worsening, and a ΔT0-T1 equal to zero no change between the two assessments. In the case of the GAF (ΔT0-T1) we inverted the signs + and – because higher scores in GAF indicate a better outcome.

Then, to test the differences between DSMs in the change (ΔT0-T1) of CGI-S, GAF and HoNOS total scores over time, we conducted a multivariate analysis of variance (MANOVA), with DSMs as the between-subjects factor.

The relationship between the rank of the percentage of improvement in staff resources and the rank of improvement in mean scores of the HoNOS, CGI-S and GAF for the various DSMs was examined by means of the Spearman correlation rank test.

### Ethical aspects

Each subject in the study was identified with a code number not ascribable to their name by researchers. Informed consent for the use of anonymous data for scientific purposes was obtained from each patient. The study was approved by the Ethics Committee at Cagliari University Hospital “Azienda Mista Ospedaliero Universitaria di Cagliari”.

## Results

### Staff resources in the DSMs studied

Table 1 shows the human resources available for mental health care in the five areas studied plus the changes in the number of staff from 2008 to 2011, i.e. the period during which the study was conducted and one year before. In all areas studied human resources were below the standards suggested by the Italian Ministry of Health. DSM1 and DSM7 were seriously deprived of resources, although in DSM7 staff resources increased by 21% during the period considered [10]. To compare the resources across the various DSMs it appeared preferable not to include the staff working in hospital wards as some DSMs (6 and 4) do not have psychiatric units at general hospitals and thus their patients are hospitalized in inpatient facilities of other DSMs (8 and 7).

**Table 1:** Staff per 1500 adult inhabitants in the Sardinian Collaborating Areas (DSMs) (Italian standard suggested by the Ministry of Health is 1 for each 1500 inhabitants)

**Sardinian Administrative areas**	**1 Sassari**	**4 Lanusei**	**6 Sanluri**	**7 Carbonia-Iglesias**	**8 Cagliari**
Total staff at the end of the study	0.65	0.70	0.73	0.55	0.78
Total staff without persons working in the hospital at the end	0.52	0.70	0.73	0.42	0.68
Total staff without persons working in the hospital at the start	0.52	0.58	0.73	0.33	0.66
% of increasing during follow-up	0	19.2%	0	21%	7%

### Socio-demographic and clinical characteristics of patients

Of a total of 309 patients who had been recruited 259 (83.8%) completed the study. Comparison between patients who participated in the study and those who did not yielded no statistically significant differences regarding gender, sex or DSM of provenance. Ninety-nine patients (38.2%) originated from the three CSMs of DSM8, 60 (23.2%) patients from DSM7, 41 (15.8%) patients from DSM4, 20 (7.7%) from DSM6 and 39 (15.1%) patients from DSM1. Among those who did not complete the study 18 (5.8%) refused to give their consent to participate and 32 (10.4%) were lost to follow-up. The socio-demographic and clinical characteristics of the study sample are illustrated in Table 2.

**Table 2:** Characteristics of the sample

**Variable**	**Descriptive statistics**
N (%) sample	259 (100%)
Age (years)	mean ± sd = 45.95 ± 12.197
	median = 44
Gender	
Male	N = 186 (71.8%)
Female	N = 73 (28.2%)
Marital Status	
Unmarried	N = 217 (83.8%)
Married	N = 21 (8.1%)
Separated/divorced	N = 18 (6.9%)
Widowed	N = 3 (1.2%)
Parenthood status	
Yes	N = 53 (20.5%)
No	N = 168 (64.9%)
Not reported	N = 38 (14.7%)
Housing	
Own home	N = 169 (65.3%)
Protected group house	N = 52 (20.1%)
Not reported	N = 38 (14.7%)
Employment status	
Retired/legally disabled	N = 140 (54.1%)
Unemployed	N = 73 (28.2%)
Employed	N = 46 (17.8%)
Education	
5 years	N = 35 (13.5%)
8 years	N = 165 (63.7%)
13 years	N = 49 (18.9%)
18 years	N = 5 (1.9%)
Not reported	N = 5 (1.9%)
ICD 10 diagnosis	
Schizophrenia	N = 167 (64.5%)
Affective psychosis	N = 92 (35.5%)
In treatment period (years)	mean ± sd = 10.61 ± 8.657
	median = 9
In treatment period ≥ 10 years	
Yes	N = 100 (38.6%)
No	N = 121 (46.7%)
Not reported	N = 38 (14.7%)
Current treatment	
Drugs	N = 101 (39%)
Integrated (drugs + psychotherapy and/or	N = 158 (61%)
Rehabilitative intervention)	

### Outcome indicators during follow-up time

Table 3 reports means and standard deviations of CGI-S, GAF and HoNOS (total score) at both time points separately for the various DSMs. It also shows the means and standard deviations of the differences between scores obtained at T0 and T1 (ΔT0-T1).

**Table 3:** Means (and standard deviations) for the CGI-S, GAF and HoNOS scale scores at T0 and T1 plus differences between both assessments (ΔT0-T1) by DSMs

		**CGI-S**			**GAF**			**HoNOS total**		
		**Mean (sd)**			**Mean (sd)**			**Mean (sd)**		
**DSM**	n	**T0**	**T1**	**Δ T0-T1**	**T0**	**T1**	Δ T0-T1*	**T0**	**T1**	**Δ T0-T1**
1. Sassari	39	5,05 (1,337)	5,03 (1,347)	,03 (,160)	31,85 (19,344)	35,38 (20,140)	3,54 (3,783)	24,56 (7,447)	22,69 (7,171)	1,87 (2,054)
4. Lanusei	41	4,78 (,791)	4,78 (,791)	,00 (,000)	46,71 (11,841)	48,98 (13,499)	2,27 (4,626)	12,15 (5,885)	10,76 (5,243)	1,34 (2,565)
6. Sanluri	20	4,55 (,887)	4,60 (,883)	-,05 (,224)	62,85 (13,188)	62,85 (13,188)	,00 (,000)	9,20 (3,708)	9,05 (3,591)	,15 (,489)
7. Carbonia-Iglesias	60	5,02 (1,761)	4,02 (1,432)	1 (1,276)	50,07 (19,409)	56,87 (17,404)	7,13 (14,230)	19,67 (9,730)	15,60 (9,374)	4,07 (5,313)
8. Cagliari	99	4,65 (1,248)	4,38 (1,323)	,26 (,599)	51,60 (16,038)	53,98 (15,129)	2,38 (5,283)	17,54 (8,397)	15,78 (7,766)	1,74 (4,174)

*In GAF (Δ T0-T1) we invert the signs + and –.

**Figure 1:**
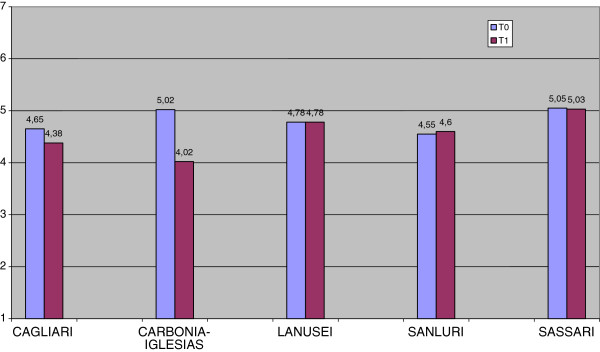
Severity of Illness (CGI-S) at T0 and T1 by DSMs.

**Figure 2:**
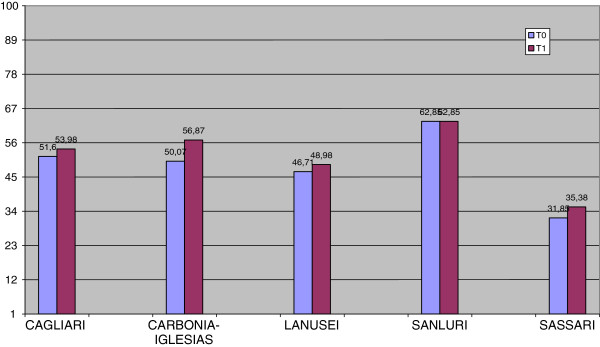
Global functioning (GAF) at T0 and T1 by DSMs.

**Figure 3:**
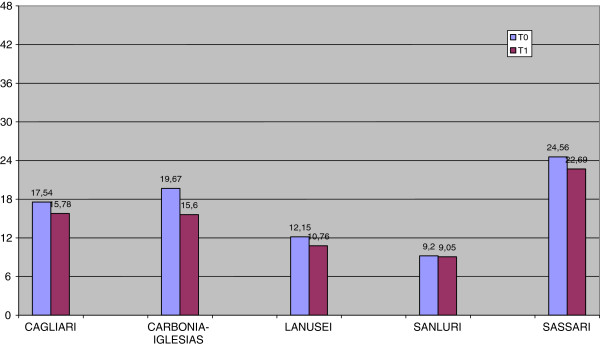
Problems related to illness (HoNOS) at T0 and T1 by DSMs.

As shown in Figures 1, 2 and 3, MANOVA revealed at T0 significant differences (F (11.324), p = 0.000) between the DSMs in the level of outcomes. Even though the severity of illness (CGI-S) was the same in all DSMs (F (1.269), p = 0.282), global functioning (GAF) was significantly better and problems related to illness (HoNOS) significantly fewer in DSM6, respectively (F (14.540), p = 0.000; F (18.674), p = 0.000).

As concerns the improvement of outcomes during follow-up, MANOVA showed a significant difference between DSMs (F (5.932), p = 0.000). DSM7 (Carbonia-Iglesias) showed the greatest improvement and DSM6 a relative stability (CGI-S: F (18.296), p = 0.000; GAF: F (4.826), p = 0.001; HoNOS: F (5.792), p = 0.000) in all outcome measures. The two DSMs (6 and 4) without psychiatric units in general hospitals were those showing greater resources in community health care services: 0.67/1.500 inhabitants against 0.57/1.500 (DSM 1,7,8) at T0 (χ2 = 45.1, 1gf, P < 0.0001) and 0.72/1.500 inhabitants against 0.62/1.500 (DSM 1,7,8) at T1 (χ2 = 58.7, 1gf, P < 0.0001). These DSMs had a better outcome on the HoNOS total score (10.19 ± 4,70 against 17.08 ± 8.12, F = 39.75, Df 1,257,258, P < 0.0001) but not with regard to CGI-S (4.70 ± 0.82 against 4.40 ± 1.35, F = 2.70, Df 1,257,258, P = 1.02) or GAF (53.53 ± 13.66 vs 51.19 ± 15.80, F = 1.088, Df 1,257,258, P = 0.298.

### Correlation between improvement in outcome measures and increase in resources

Table 4 shows the correlation between the improvement in human resources during and one year before the follow-up and improvement in outcome indicators. As can be seen, an increase in staff was positively correlated with an improvement in the total score of the HoNOS. A similar correlation was not found with the GAF and CGI-S.

**Table 4:** Increase in staff resources and changes in outcome measures in the five DSMs studied

	**% Increase in staff**	**% Improvement in honos scores**	**% Improvement in CGI scores**	**% Improvement in GAFscores**
1. Sassari	0	8.2	0.6	10.0
4. Lanusei	19.2	12,4	0	4.6
6. Sanluri	0	0	−1.1	0
7. Carbonia-Iglesias	21	26,1	24.8	12.5
8. Cagliari	7	11,0	5.9	4.4
Spearman correlation coefficient		SRRC0.975 P < 0,01	SRRC = 0.625 n.s.	SRRC = 0.575n.s.

## Discussion

The study shows that despite a heated ideological debate about how to organize mental health services in Sardinia, public services remain very resource-poor, at least in terms of human resources. At the start as well as at the end of the study, none of the DSMs that participated in the study, providing two thirds of all community care for the Sardinian Region, reached the number of professionals per 1,500 inhabitants (1.0/1.500) recommended by the Italian Ministry of Health Guidelines as indicative of good quality of care [16].

If one compares our data with those reported by the National Institute of Health, the gap in terms of available staff is even larger. The human resources were in fact 1.8 per 1,500 inhabitants in Trieste, 1.2 in Arezzo and 1.6 in South Verona [17]. In contrast, the number of patients cared for by the public mental health care system in the Region of Sardinia in 2006, 180 patients per 10,000 inhabitants on average in the whole region, is higher than that reported by the National Institute of Health in 2004 in Trieste (151 per 10,000), Arezzo (149 per 10,000) and much higher than that in South Verona (67 per 10,000) [17]. These differences may be due to non-standardized collection of routine data but it is also understandable that in a poor region such as Sardinia people have less opportunity to turn to private agencies outside of the Italian national health system than in the rich regions of northern Italy.

Both the health managers inspired by the Trieste model who governed the public health services up to 2009 and the opponents of that model have failed to provide citizens with a minimum of resources required for an acceptable level of quality of care. The inadequacy of the network of community mental health services is also indicated by the fact that the resources are insufficient even in the DSMs that have psychiatric units in general hospitals. Our data suggest that hospital treatment remains still relevant in a system of care that should primarily be focused on community care (as stated in Law 180).

Across the whole study sample, the poverty of resources is one of the key factors associated with high levels of illness severity and low levels of functioning and social outcomes (particularly those measured by the Honos) at follow-up. In fact, the level of illness severity observed in our study is even more pronounced than that found with similar instruments in national [18] and international [19] samples of patients with psychoses. In the study of Parabiaghi et al. [18], carried out in 2003 in nine communities mental health services in Italy, a large cohort of 2059 patients was monitored over a one-year period. Patients were subdivided according to length of time in treatment and diagnosis. Of the 1431 patients in treatment by the public health care network for at least two years, about 80% had been given the diagnosis of schizophrenia, bipolar disorder or personality disorder. This group showed a mean Honos score of 11.36 ± 6.21 with a mean of improvement of 1.32 (SD 0.21) at the end of the follow-up. This was similar to our observations in two smaller areas (DSM 6 and DSM4). All other patients showed lower levels of functioning, particularly those from the two metropolitan areas (DSM1 and DSM8), which account for over half of the population of Sardinia.

Our findings also confirm, twenty years later, the results of the European ERGOS study conducted in 1994. In this study, the sample of patients with schizophrenia attending the public mental health center in Cagliari (corresponding to the present-day DSM8) were those with the highest level of unmet social needs among the 12 European centres participating in the research [9]. In the same study it was found that the Sardinian center had very poor staff resources. It should be noted that with regard to unmet clinical needs the patients of the Sardinian center were considered within the European range.

Also in our study patients’ scores on the CGI and GAF did not show an association with those on the HoNOS.

Thus, the present study appears to confirm the result of the Ergos study that poverty of staff resources influences social functioning and needs of patients more than clinical outcomes. The CGI is in fact a simple clinical scale, and the GAF, although a general measure of functionality, includes clinical outcome with substantial weight, while the score of the HoNOS, also considering a clinical factor, is much more determined by precise factors related to social and work functioning.

From this point of view, an important observation concerning staff resources is the comparison relating only to medical staff. The data given by the Region of Sardinia for the whole region almost coincidentally with the start of the study is in fact comparable to that seen in our collaborating centers for all of the professionals (0.68 × 10,000 inhabitants) [20] but if we look at the data relating only to psychiatrists in the observed rate, Sardinia (0.19 per 10,000 inhabitants) is in the range of one of the centers of excellence previously mentioned (0,21 × 10,000 inhabitants in Trieste, 0.195 in Verona; 0,16 in Arezzo), it is therefore likely that the worst social outcomes (compared with clinical outcomes) detected by our study are dependent on the fact that the major deficiencies of staff are related to non-medical staff [17,20].

The quality of mental health services is defined on the basis of structure, process and outcome indicators [21-23]. The Continuous Quality Improvement model points out the interplay between these three components. In this sense, outcomes could be considered as being indirectly linked to processes and structures [24,25]. Nevertheless, they are measured and recorded less frequently than structure and process indicators, often because their measurement is considered to be more expensive [26]. On the other hand, assessment of outcome indicators is relatively easy, given the availability of clinical data sources across providers [21] although it can be argued that the outcomes need to be clarified according to social functioning or simple symptom reduction.

The results of our study indicate that the poor outcome in general health and social functioning may result from the paucity of staff resources although we did not measure the quality of staff, which needs to be explored in future studies. This conclusion is supported by the comparison of results from our sample with those from the aforementioned national and international samples as well as by comparisons within our sample, showing that better staffed community mental health centers (DSM4 and 6) are the ones where a higher level of social functioning was measured with the HoNOS. In addition, an increase in staff was positively correlated with an improvement of the HoNOS score. A similar correlation was not found with the scores of the CGI and the GAF. Future research needs to explore the quality of interaction between staff and patients as well as the quality of the environment in which such encounters take place. Furthermore, the numbers in our study are small and it may be that those who had worse outcomes were also the most difficult to engage, thus making our findings even more alarming. Human resources are important but are also the most expensive part of the health care budget. Policy makers thus need to be convinced to invest in staff and their training so that the most vulnerable individuals can be looked after and their needs met.

The discussion of models of mental health care is a prerequisite for establishing a coherent system of care [27]. However, if the debate is not followed by an adequate availability of resources it will not give answers to the needs of patients. It is also unlikely that the resources that have not been made available in times of economic well-being will be allocated to mental health care under conditions of the current economic crisis which calls into question the entire system of welfare in Europe.

## Conclusions

The study shows that mental health services provided in the public sector in Sardinia are still very resource-poor, at least in terms of human resources. Our findings suggest that mental health service resources influence outcomes as regards the social functioning of users. We urge policy makers to take these observations into account when planning future services.

### Limitations

Our findings are to be seen in the light of the limitations of the study. To be mentioned are the relatively small size of the patient sample and the use of rather simple instruments for measuring outcome, which may be more suitable for monitoring routine care than for a research project. Another limitation is the relatively brief observation period (1 year). It would certainly have been interesting to collect information on the organization of staff and patient care, but the resources are so few and sub-standard that even a coarse measure as the mere amount of available human resources is of interest.

## Competing interests

The authors declare that they have no competing interests.

## Authors’ contributions

MGC had the idea of the study, participated in the design and coordination of the study, in the acquisition and analysis of the data and drafted the manuscript. MCA and DB participated in the design and coordination of the study, in the acquisition and analysis of the data and drafted the manuscript. FS, FT and RP participated in the coordination of the study, in the acquisition and analysis of the data and drafted the manuscript. AP, EP, GM, MP, EP, MFM and GT participated in the acquisition of the data and drafted the manuscript. DM participated in data analysis and drafted the manuscript. All authors read and approved the final manuscript.

## Pre-publication history

The pre-publication history for this paper can be accessed here:

http://www.biomedcentral.com/1471-244X/13/333/prepub
